# JUSTFAIR: Judicial System Transparency through Federal Archive Inferred Records

**DOI:** 10.1371/journal.pone.0241381

**Published:** 2020-10-26

**Authors:** Maria-Veronica Ciocanel, Chad M. Topaz, Rebecca Santorella, Shilad Sen, Christian Michael Smith, Adam Hufstetler

**Affiliations:** 1 Department of Mathematics, Duke University, Durham, NC, United States of America; 2 Institute for the Quantitative Study of Inclusion, Diversity, and Equity, Williamstown, MA, United States of America; 3 Department of Mathematics and Statistics, Williams College, Williamstown, MA, United States of America; 4 Division of Applied Mathematics, Brown University, Providence, RI, United States of America; 5 Department of Mathematics, Statistics, and Computer Science, Macalester College, St. Paul, MN, United States of America; 6 Department of Sociology, Berea College, Berea, KY, United States of America; 7 Department of Electrical and Computer Engineering, Duke University, Durham, NC, United States of America; University of Vermont, UNITED STATES

## Abstract

In the United States, the public has a constitutional right to access criminal trial proceedings. In practice, it can be difficult or impossible for the public to exercise this right. We present JUSTFAIR: Judicial System Transparency through Federal Archive Inferred Records, a database of criminal sentencing decisions made in federal district courts. We have compiled this data set from public sources including the United States Sentencing Commission, the Federal Judicial Center, the Public Access to Court Electronic Records system, and Wikipedia. With nearly 600,000 records from the years 2001—2018, JUSTFAIR is the first large scale, free, public database that links information about defendants and their demographic characteristics with information about their federal crimes, their sentences, and, crucially, the identity of the sentencing judge.

## Introduction

In the United States, the public’s right to access court proceedings and court records is frequently debated and litigated [1]. However, with respect specifically to criminal trials, the public’s right of access is unambiguous. A 7-1 majority of the Supreme Court affirmed this right in the case *Richmond Newspapers, Inc. v. Virginia*, 448 U.S. 555 (1980). The opinion of the plurality held that “the First Amendment guarantees of speech and press, standing alone, prohibit government from summarily closing courtroom doors.” Federal court proceedings may be closed in special cases involving classified information, trade secrets, ongoing investigations, or other complications. By default, however, most proceedings are open [2, 3].

While members of the public have the right to attend criminal trials, they are not necessarily well-positioned to exercise this right. Any individual could in principle choose to attend a federal criminal court proceeding and observe the defendant, the judge, the crimes allegedly committed, the evidence provided, the verdict, and, if applicable, the sentence given. However, no individual can attend the hundreds of thousands of proceedings that take place in federal courts every year. Stated differently, the public does not have access to high quality, large-scale information about the federal criminal justice system, and therefore, much of what happens in criminal courts remains opaque. The opacity of the federal criminal court system impedes the public in a number of ways, including in its ability to assess sentencing equity. As we explain later, while federal sentencing guidelines that recommend appropriate penalties exist, they are not binding. Judges have broad discretion in their choice of sentences.

Sentencing disparity is an active area of research in legal scholarship. One stream of this research investigates associations between the severity of sentences and demographic characteristics of defendants. A study of over 77,000 federal offenders finds, after controlling for numerous factors, that judges give longer sentences to individuals who are Black, male, or low-income as compared to members of other racial, gender, and income groups [4]. More recent work confirms sentencing gaps biased against men and Black individuals [5–7]. For example, Rehavi and Starr construct a multi-agency-based dataset that focuses on offender and offense information. Their data includes almost 37,000 cases of black and white male US citizens who were arrested for violent, property/fraud, weapons, and public order offenses and referred to federal prosecutors for potential prosecution between fiscal years 2006 and 2008. This dataset provides very detailed demographic and arrest/prosecution information for the offenders, but is not linked to judge identity or information. A second stream of research focuses not on characteristics of defendants, but rather, on those of judges [6, 8–10]. For instance, [10] dissects the role of political affiliation in federal criminal cases and finds that as compared to Democratic-appointed judges, Republican-appointed judges give longer sentences to defendants who are Black or male.

While this second, judge-focused stream of research does examine associations between judge demographics and sentencing disparities, it does not shed light on the sentencing decisions of individual judges. As the authors of [8] explain, “The unavailability of judge data is one of the most frustrating aspects of the study of federal sentencing and has significantly impeded scholarly evaluation.” The U.S. government does publish certain data sets related to federal criminal sentencing, available from the United States Sentencing Commission. However, as [8] also notes, “the Commission has refused to identify the sentencing judge in the data.”

Despite the U.S. government’s decision not to identify sentencing judges, studies like [6, 8–10] have been possible because the investigators have assembled their own databases to identify judges. The painstaking work of [8] manually assembles a database of 2,265 records using information from the United States Sentencing Commission and the Public Access to Court Electronic Records System. However, while the data set does link individual judges with sentencing decisions, no individual judges are discussed in [8], nor is the data set publicly accessible. The studies in [6, 9, 10] are much larger scale, involving hundreds of thousands of sentencing decisions, each linked to information about the sentencing judge. To build these databases, the authors combine data from government databases with data from a proprietary source, the Transactional Records Access Clearinghouse (TRAC), which provides information about judges’ sentencing habits obtained via Freedom of Information Act requests. This dataset consisted of almost 547,000 cases linking federal sentencing data with judge information for defendants sentenced between 1999 and 2015 [10]. To connect between defendant and crime characteristics and sentencing judge information, the authors matched records in these databases based on “district court, sentencing year, sentencing month, sentence length in months, probation length in months, amount of total monetary fines, whether the case ended by trial or plea agreement, and whether the case resulted in a life sentence” [10]. However, TRAC’s assembled data is proprietary and is governed by a data usage agreement [11]. Presumably for these reasons, the data sets used in [6, 9, 10], which incorporate information from TRAC, are not public, and these manuscripts do not disclose information about the sentencing decisions of individual judges.

In summary, the public has a constitutional right to observe criminal proceedings, and in principle could tabulate the sentencing decisions of every federal judge. However, due to practical constraints, individuals cannot exercise this right on a large scale. The U.S. government publishes data about criminal sentencing, but this data does not identify the judge associated with any particular sentencing data. Past legal scholarship has linked sentencing decisions with individual judges, but the proprietary data source used prohibits the identification of these judges.

To remedy this situation, we present JUSTFAIR: Judicial System Transparency through Federal Archive Inferred Records, a database of criminal sentencing decisions made in federal district courts. We have compiled this data set from public sources including the United States Sentencing Commission, the Federal Judicial Center, the Public Access to Court Electronic Records system, and Wikipedia. With nearly 600,000 records from 2001—2018, JUSTFAIR is the first large-scale, free, public database that links information about defendants and their demographic characteristics with information about their federal crimes, their sentences, and crucially, the sentencing judges. Our publicly-available database extends the one in [10] to fiscal years 2002-2018 and uses similar variables (district court, sentencing date, prison sentence length, probation length, fine amount, offense codes, etc.) to match records in the appropriate public sources.

The rest of this paper is organized as follows. In Legal Background, we provide a layperson’s overview of the federal court system and a brief history of federal criminal sentencing. In Data Sources and Preprocessing, we describe in detail our procedures for acquiring data from four sources and for preparing that data for merging. In Data Consolidation, we explain how we merge United States Sentencing Commission data first with Federal Judicial Center data to obtain court docket numbers, then with the Public Access to Court Electronic Records system to obtain the initials of the sentencing judge for each case, and finally, with information from Wikipedia (as well as from the Federal Judicial Center) to obtain the full name of each judge. Finally, we conclude with a brief summary of JUSTFAIR and some directions for future work.

## Legal background

### Structure of the Federal Judicial System

For a layperson’s review of the federal court system, see, *e.g*., [12]. In our work, our primary concern is the principal federal trial court system, which comprises 94 district courts. Each U.S. state has one or more federal district courts. For instance, Alaska has one district, whereas California, New York, and Texas each have four districts. Additionally, the District of Columbia and Puerto Rico each have one district court. Altogether, the U.S. states, District of Columbia, and Puerto Rico account for 91 of the 94 aforementioned courts. The remaining three courts are associated with the territories of Guam, the Northern Mariana Islands, and the U.S. Virgin Islands. While these three territorial courts are established in a different part of the Constitution than the 91 districts, they have similar jurisdiction, and so it is common to refer to them as district courts. Nonetheless, there are some differences between these territorial courts and the other district courts. Excluding the three territorial courts, each district also has a specialized bankruptcy court. Additionally, there are two special courts that have nationwide jurisdiction, namely the U.S. Court of Federal Claims and the U.S. Court of International Trade.

Decisions made by judges in the 94 district courts can be appealed. Each district court belongs to one of twelve circuits, and each circuit has an appeals court. A thirteenth circuit, the Federal Circuit, hears appeals for certain special types of cases. The Supreme Court sits above the federal circuit courts of appeals. While the U.S. Constitution and federal laws mandate special types of cases that the Supreme Court must hear, much of the Supreme Court’s case load is chosen by the court itself, whenever at least four justices agree to hear a case. One important role of the court is to resolve situations where two or more circuit courts of appeals issue legal rulings that are in conflict.

Excluding judges on the three territorial courts, federal district judges are chosen as specified in Article III of the Constitution. That is to say, they are appointed by the president of the United States and must be confirmed by a vote of the Senate. These Article III judgeships are lifetime appointments. Once judges are confirmed, they cannot be removed from office except through impeachment by the House of Representatives followed by conviction in the Senate. Judges in the three territorial courts are not governed by Article III of the Constitution, and thus do not have lifetime appointments.

Federal district courts also have magistrate judges. A district magistrate judge is chosen not by the president, nor by Congress, but rather, by a majority vote of the federal judges in the district. Magistrate appointments last for eight years and are renewable. The specific duties of magistrate judges are not prescribed by federal law. Each district court has the authority to delegate to its magistrates “additional duties as are not inconsistent with the Constitution and laws of the United States” [13].

### Federal criminal sentencing

For an overview of federal sentencing, see [14]. Prior to 1987, federal district judges had the discretion to impose, nearly without restriction, the criminal sentences they deemed appropriate. Put concretely, two individuals with similar criminal histories who were convicted of committing similar crimes under similar circumstances could receive vastly different sentences depending on the outlook of the presiding judge.

However, in 1984, Congress passed the Sentencing Reform Act as part of a larger piece of legislation, the Comprehensive Crime Control Act. The Sentencing Reform Act established the U.S. Sentencing Commission which, in 1987, formally adopted federal sentencing guidelines in order to mitigate sentencing disparities. These guidelines set uniform sentencing policies for felonies and for the most serious class (Class A) of misdemeanors. The guidelines are a detailed set of instructions for algorithmically calculating the appropriate range of a sentence. The algorithm accounts for the type of crime, aggravating and mitigating factors, criminal history, and more.

These sentencing guidelines were originally interpreted as mandatory. However, in *United States v. Booker*, 543 U.S. 220 (1985), the Supreme Court ruled that these sentencing guidelines cannot be mandatory and must be considered merely as advisory. Today, federal district court judges are required to calculate the sentence recommended by the guidelines, but they can still issue a different sentence that they deem appropriate, subject to mandatory minimum sentences when applicable.

Generally speaking, magistrate judges do not impose criminal sentences. The primary conditions under which a magistrate judge might impose a criminal sentence are when the conviction is for a Class A misdemeanor and the defendant waives their right to be sentenced by a federal district judge.

## Data sources and preprocessing

### Overview of data sources

One of our primary goals is to facilitate study of the criminal sentencing decisions of individual federal district judges. JUSTFAIR includes information about federal criminal cases including:

- demographic characteristics of the sentenced individual,
- sections of the law relevant to the conviction,
- factors influencing the recommended sentence,
- components of the sentence, including (potentially) a fine, probation, and prison time,
- sentencing date,
- federal district of the court, and
- name, time period of service, appointing president of the sentencing judge, as well as other background information.

To assemble JUSTFAIR, we consolidate data from five sources:

- the United States Sentencing Commission Database,
- the Federal Judicial Center Integrated Database,
- the Public Access to Court Electronic Records system,
- Wikipedia, and
- the Federal Judicial Center Biographical Directory of Article III Federal Judges.

**Fig 1:**
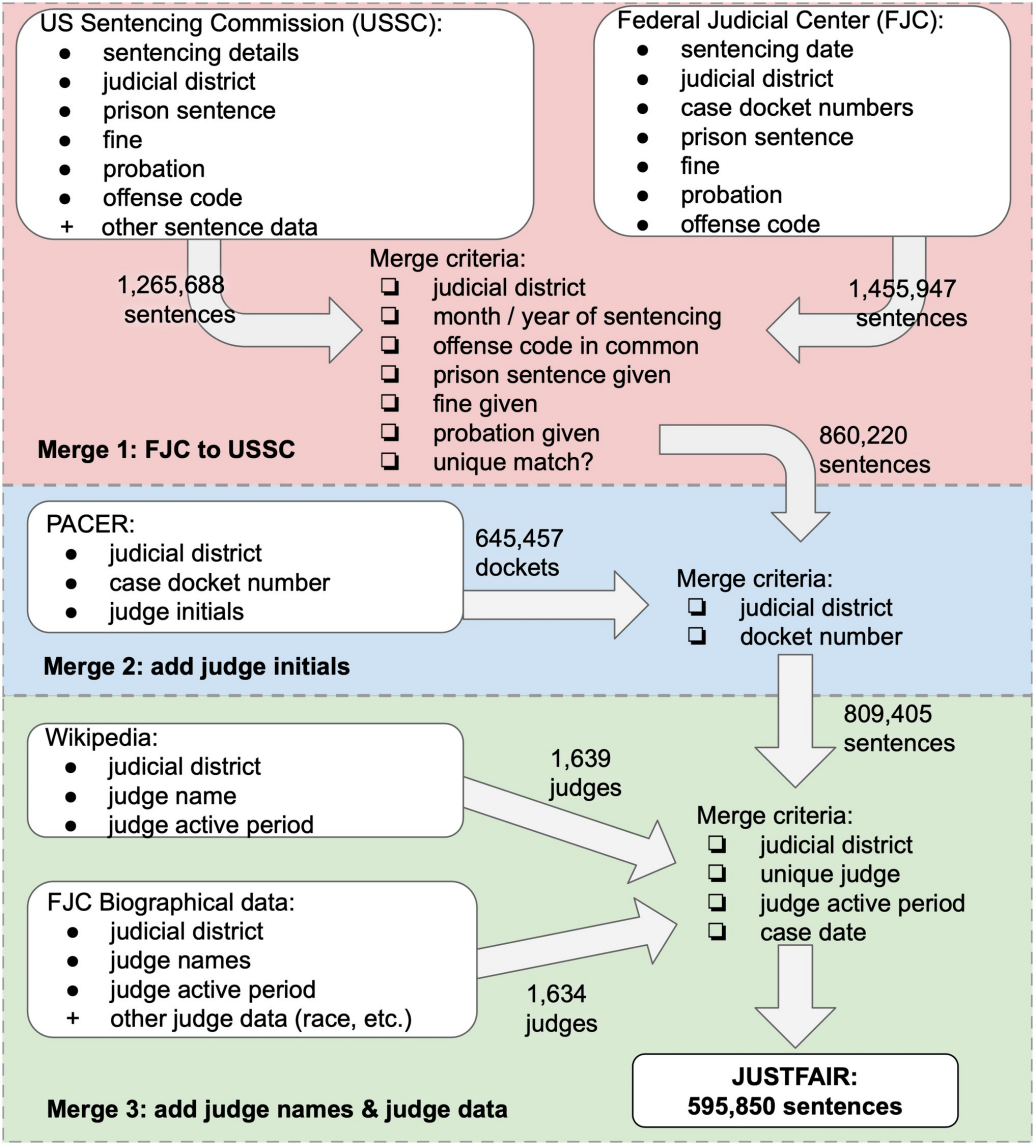
JUSTFAIR data pipeline. We combine information from the United States Sentencing Commission (USSC), the Federal Judicial Center (FJC), the Public Access to Court Electronic Records System (PACER) and Wikipedia in order to create our database, Judicial System Transparency through Federal Archive Inferred Records (JUSTFAIR). With nearly 600,000 federal district court records from 2001—2018, JUSTFAIR is the first large-scale, free, public database that links information about criminal defendants and their demographic characteristics with information about their federal crimes, their sentences, and crucially, the sentencing judges.

Fig 1 illustrates the data pipeline used to create JUSTFAIR. First, we obtain detailed information about criminal cases and sentences (pink section of figure) from the United States Sentencing Commission database. This database includes demographic information about convicted individuals and detailed information about sentences given. However, while this database provides the sentencing date and federal judicial district of the case, it does not identify the sentencing judge. To obtain information about the sentencing judge requires a case docket number. We obtain these case docket numbers from the Federal Judicial Center Integrated Database and merge them with the United States Sentencing Commission database (pink section of figure). Then, from the Public Access to Court Electronic Records system, we retrieve the initials of the sentencing judge associated with each docket number (blue section of figure). In order to map judge initials to an identified judge, we retrieve judge names from two different sources (green section of figure). From Wikipedia, we scrape the names of current and former federal district judges and extract their initials. We also use the Federal Judicial Center Biographical Directory of Article III Federal Judges as an alternative means to obtain the names of current judges as well as their demographic, education, and nomination information. In the following subsections, we describe our five data sources in greater detail, and we explain the procedures we use to preprocess the data and merge it to create the JUSTFAIR database.

### United States Sentencing Commission

The United States Sentencing Commission (USSC) is an independent agency of the federal judicial system created by Congress via the Sentencing Reform Act of 1984. USSC’s mission includes the directive “to collect, analyze, research, and distribute a broad array of information on federal crime and sentencing issues, serving as an information resource for Congress, the executive branch, the courts, criminal justice practitioners, the academic community, and the public” [15]. In service of this mission, USSC maintains publicly accessible data sets, including files which provide information about sentences given to individuals in federal district courts. We refer to these as individual offender files (IOFs) to distinguish from other public files that tabulate information on corporate offenders [16]. USSC provides a detailed description of IOF contents in their code book [17].

Seventeen IOFs are available, one each for the fiscal years 2002—2018 (inclusive). These files come in.dat format, and range in size from 1.19 gigabytes to 4.07 gigabytes, with a median size of 2.02 gigabytes. In total, the IOFs comprise 35.08 gigabytes. We convert these files to.csv format for convenience. Each file has tens of thousands of records (rows), ranging from approximately 64,000 to approximately 86,000 with a median of approximately 73,000. Each file also has thousands or tens of thousands of variables (columns). The differing numbers of variables from file to file are due to changes in sentencing policies and record keeping over time, as detailed in [18–22]. As we will explain momentarily, a majority of these columns are mostly empty.

To make the data set amenable to study by the public, we reduce it to a more tractable size. First, we construct the set of column names in each IOF and we intersect these sets (ignoring case, which is inconsistent across years). There are 2,384 variable names that are shared across all 17 files. Additionally, there are 16 variables that we retain for possible downstream analysis: SENTDATE, SENTMON, SENTYR, SENTTOT0, BOOKER2, BOOKER3, BOOKERCD, BOOKPOST, DEFCONSL, OFFTYPE2, OFFTYPESB, and NWSTAT1—NWSTAT5. We eliminate all other variables. We then examine the amount of data present in the retained variables. Many of these columns consist primarily of missing data. We retain variables that have at least 25% of data present, a cutoff chosen by inspection of the histogram of the percentage of data present in each column (not shown). After eliminating the mostly-empty columns from each file, we again intersect variable names across all IOFs. In the previous two steps, however, we force inclusion of the 16 variables enumerated above. Altogether, we identify 163 columns to retain. We reduce all IOFs to these shared columns and concatenate the 17 files. The result is a 0.5 gigabyte.csv file with 1,265,688 records and 163 variables. In our data set, we retain the variable USSCID which is a unique identifier in the USSC database. Because we retain this identifier, any user wishing to recover variables we eliminated from the raw IOFs can do so in a straightforward manner.

We make several modifications to our USSC data. First, we reconcile a change in the coding of sentencing dates. For the years 2001—2003, the month and year when each sentence was given are coded in one variable called SENTDATE. From 2004 onwards, the month and year are stored separately in variables SENTMON and SENTYR. We extract the months and years from SENTDATE in the 2001—2003 data to have a consistent format for dates.

Second, we create modified versions of the variable SENTTOT. This variable stores the total prison sentence given in months. Because of this choice of units, the values of SENTTOT are not necessarily integers. Later, we merge our USSC data with data from the Federal Judicial Center Integrated Database by matching on several variables, including sentence length. However, in the latter database, all values of prison sentence length are integers. Therefore, to facilitate merging, we create variables roundSENTTOT and floorSENTTOT, where the former rounds SENTTOT to the nearest integer and the latter truncates any fractional portion of SENTTOT.

Third, we reconcile SENTTOT with another variable, TOTPRISN (total months of imprisonment, not including time served). TOTPRISN may take on a number of special values, including 9996, and 9998, which correspond to life imprisonment and the death penalty, respectively. We carry these values over to SENTTOT to retain information about these penalties.

Fourth, we clean SENTTOT for certain records. In particular, we note 157,484 records for which SENTTOT has missing data but a closely related variable, SENTTOT0 has the value of zero. According to the USSC data codebook [17], SENTTOT codes zero-length sentences as missing, while SENTTOT0 does not. Because [19] recommends using the variable SENTTOT for analysis, but because we need information about zero-length sentences, we recode SENTTOT as zero for the 157,484 aforementioned records. We also create a new variable, SENTTOTFLAG, which flags the records that have been recoded.

Fifth, we create modified versions of the variables NWSTAT1—NWSTAT5. These variables record each portion of the U.S. legal code under which a defendant has been convicted. These codes include, potentially, the relevant title, section, and subsection of the U.S. legal code. For instance, one code in the data set is 181956A1, which refers to a subsection of U.S. Code Title 18, Section 1956 on money laundering. We truncate the NWSTAT codes to include only the title and section information so that, for example, 181956A1 becomes 181956. We refer to our truncated variables as USSCcode1—USSCcode5, and these will validate matching with other data sources downstream.

Finally, we set any empty fields in the database to NA (short for “not available”) to represent missing data. After the preprocessing steps described above, our final USSC data set has 1,265,688 records and 171 variables.

### Federal Judicial Center integrated database

The Federal Judicial Center (FJC) is an agency of the U.S. federal judicial system, established in 1967 with a tripartite mission that includes “research and study of the operation of the courts.” To this end, the FJC maintains and provides public access to an Integrated Database [23] consisting of seven files with information on civil cases, criminal cases, appeals cases, and bankruptcy cases. The criminal cases are spread across two files, the second of which covers the time period from 1996 to present. We download this data, which FJC provides as a 2.7 gigabyte tab-delimited text file [24]. The raw file comprises 4,783,129 records and 144 variables. FJC provides a detailed description of file contents in their code book [25].

We make several modifications to the FJC data that we download. First, we create new variables for sentencing date. The raw variable SENTDATE encodes the day, month, and year of sentencing. From it, we extract two new variables, SENTMON and SENTYR, which store the month and year separately for commensurability with USSC data downstream.

Second, because USSC data begins in calendar year 2001, we restrict our FJC data to this same time period. This restriction drastically reduces the number of records. In fact, the raw FJC file not only includes data from prior to 2001, but it includes over three million records whose sentencing dates are listed as January 1, 1900. We exclude these records. By retaining FJC records with sentencing dates in 2001 or later, we reduce the data set to 1,455,947 records.

Third, we address issues with the coding of the federal judicial district for each record as stored in the variable DISTRICT. The coding of judicial districts in FJCIDB differs from that in USSC for eight districts, namely the Middle District of Florida, the Southern District of Florida, the Northern District of Georgia, the Middle District of Georgia, the Southern District of Georgia, the Eastern District of Louisiana, the Middle District of Louisiana, and the District of Alaska. In the FJC Integrated Database, these are coded as 3A, 3C, 3E, 3G, 3J, 3L, 3N, and 7-, respectively. We replace these codes with the numbers 30—35, 96, and 95, respectively, to be commensurate with the DISTRICT variable in USSC. Additionally, values of -8 signify missing information, and we replace these with NA.

Fourth, we recode the sentencing variable PRISTOT, an integer specifying the total prison sentence given in months. While most values are positive integers, the data also contains certain special negative values. Values of -1 signify imprisonment of less than one month and values of -2 signify that no prison sentence was imposed. We replace these values with zeros for commensurability with USSC data. Values of -3 represent sealed sentences, and we replace them with NA since it is not possible to know the sentence. Values of -4 and -5 represent life imprisonment and the death penalty, respectively. We replace these values with 9996 and 9998 to match the USSC database. There are also 38,375 records with PRISTOT equal to -8, signifying missing data. For these records, we examine the variables PRISTIM1—PRISTIM5 which give the amount of prison time to which a defendant was sentenced for up to five sections of the U.S. criminal code. For all 38,375 records, the value of PRISTIM1 is zero, and the values of PRISTIM2—PRISTIM5 are either -8 (signifying missing data) or zero. In summary, the PRISTIM variables for these records do not indicate any prison sentence, and so we set the value of PRISTOT to be zero for them. We also create a new variable, PRISTOTFLAG, which flags the records that have been recoded.

Fifth, we recode the sentencing variable PROBTOT, which specifies the number of months of probation given with a sentence. We perform this recoding in a manner very similar to that used above for PRISTOT. Values of -1 represent probation of less than one month and we replace these with zeros for commensurability with the USSC data. Values of -8 represent missing data, of which there are 353,000 occurrences. For these records, we examine the variables PROBMON1—PROBMON5 which give the amount of probation time to which a defendant was sentenced for up to five sections of the U.S. criminal code. For all 353,000 records, the value of PROBMON1 is zero, and the values of PROBMON2—PROBMON5 are either -8 (signifying missing data) or zero. In summary, the PROBMON variables for these records do not indicate any probation time, and so we set the value of PROBTOT to be zero for them. We also create a new variable, PROBTOTFLAG, which flags the records that have been recoded.

Sixth, we create ten new variables, FJDcode1—FJDcode10, which are modified versions of the variables FTITLE1—FTITLE5 and TTITLE1—TTITLE5. The first five variables record portions of the U.S. code under which a case was filed, and the latter five record portions of the U.S. code under which a case was disposed. For commensurability with the USSCcode1—USSCcode5 variables we created in our preprocessed USSC database, we eliminate punctuation from these strings and reduce them to include only the title and section of the U.S. code.

Finally, we set any empty fields in the database to NA to represent missing data. After the preprocessing steps described above, our FJC data set contains 1,455,947 records and 158 variables.

### PACER and juriscraper

Public Access to Court Electronic Records (PACER) is the records access service of the United States federal courts. This database was established in 1988 and was made publicly accessible online beginning in 2001 [26]. For each court case it contains, PACER includes a breadth of information ranging from a roster of involved parties, to chronologies of the judicial process, to scanned court documents, to, potentially, judicial opinions issued. PACER content for each district court is maintained by that court on its own website. However, there is a centralized PACER Web portal [27] with a search feature that connects to individual court sites. Currently, access to PACER records is for-fee at the rate of $0.10 per page of information accessed.

Our queries to PACER require us to have a case docket code. Constructing this code requires the variable CASLGKY, which originates in the FJC data. CASLGKY is a 15 character string of the form “CCDDOYYSSSSSTTR” where:

- Characters CC are digits specifying the federal judicial circuit of the court where the case occurred.
- Characters DD are digits or letters specifying the federal judicial district.
- Character O is a digit or letter indicating which office within the judicial district handled the case.
- Characters YY are the last two digits of the year in which the case was filed.
- Characters SSSSS represent the five-digit sequence number of the case (essentially, an ID number assigned to the case by each court).
- Characters TT are either “CR” or “MJ,” specifying, respectively, a criminal case handled by a federal district judge or one handled by a magistrate judge.
- Character R is the one-digit reopen sequence number indicating whether the record corresponds to an original court proceeding or the re-opening of a previous proceeding.

From CASLGKY we construct a federal docket number. Each docket number has the form “O:YY-CR-SSSSS” where the characters O, YY, and SSSSS are as defined above, and “CR” indicates that we are searching only for cases handled by district judges. We use our docket number and the DISTRICT variable to query Juriscraper, a tool of the FreeLaw Project [28]. Juriscraper provides an API to retrieve metadata from district court websites.

A Juriscraper query on a docket number returns a case title. This case title begins with a version of the docket number, followed by information. The version of the docket number that is returned may include additional letters that we did not submit as part of our search query. For the vast majority of districts, these are the initials of the judge (we discuss exceptions later). For example, we use Juriscraper to query the docket number 2:01-CR-00071 to the District of Maine. This is a criminal case from the second court within the district, filed in 2001, having sequence number 00071. The query returns PACER’s full case title, which is “2:01-cr-00071-DBH USA v. BLAKE (closed 01/03/2002).” The appended letters “DBH” are the initials of a judge within the district, in this case, Hon. David Brock Hornby. Some PACER case titles include two sets of initials, for example, “1:00-cr-00425-RWR-AK.” We store the first set of initials in the variable pacer_first_inits. If the second set of initials is present, we store it in variable pacer_second_inits.

In cases that have multiple defendants, a single Juriscraper query may return more than one result. In fact, we submit 744,401 queries to Juriscraper (see Merging Federal Judicial Center Integrated Database), and these queries return 3,394,237 results with 645,457 unique values of CASLGKY.

### Wikipedia

To obtain information about federal district judges, we scrape Wikipedia. We begin with Wikipedia’s page containing a master list of U.S. district courts [29], and from it, we retrieve the link to each individual court’s Wikipedia page. These individual court pages each contain tables with information about the judges currently and formerly on the court. We scrape these tables, retaining information such as judge name, lifespan, years active, and link to the Wikipedia page for the judge (if available).

Eventually, we will use information about judges’ initials from PACER records in order to infer the full name of the judge. To enable this inference, we must extract judge initials from our scraped Wikipedia data. Before performing this extraction, we clean the scraped judge names. First, we remove strings in judge names that do not contribute to the judge’s initials. These strings include Roman numerals “I” through “V” as well as “Jr.,” “Sr.,” and footnotes or numbers such as “[1]” that were present in some scraped records. Second, we deal with apostrophes in names, based on a manual investigation of judge initials within PACER. For instance, in PACER, Hon. Beverly Reid O’Connell has initials “BRO.” Thus, we eliminate all apostrophes from our Wikipedia names. Third, we deal with hyphens in names, also based on manual investigation. For instance, Hon. Martin Leach-Cross Feldman has initials “MLCF” in PACER. Thus, we replace hyphens in Wikipedia names with spaces. Fourth, we remove parenthetical nicknames appearing for some judges, such as Hon. Wilhelmina Marie (Mimi) Wright. Fifth and last, we manually edit a small number of entries. Specifically, we encode Hons. James Arnold von der Heit, John W. deGravelles, and Frederick van Pelt Brian as “JAH,” “JWG,” and “FPB,” respectively. With these data cleaning procedures complete, we extract initials from each judge’s name simply by breaking each name into space-separated tokens and retaining their first letters. We record these initials in the variable judge_inits.

Some judges have a middle name listed on their individual Wikipedia page, but not in the table on their district’s Wikipedia page. For example, Hon. Abdul Karim Kallon’s name is given simply as Abdul Kallon in the table on the Wikipedia page for the Northern District of Alabama. To account for these cases, we access all available individual district judge Wikipedia pages (linked from the tables in the courts’ Wikipedia pages) and scrape each judge’s full name. We extract initials from these names in a manner similar to that used above. This second version of a judge’s initials could be identical to the first version, could be a more complete version that incorporates additional middle names, or could be “N” for Null if that judge does not have an individual Wikipedia page (we record this in the variable judge_inits_full).

Finally, we add to our data set the federal judicial district in which each judge serves or served. There are in fact two numbering schemes for judicial district used in the USSC data, namely, the variables DISTRICT and CIRCDIST; see [17]. We add both codes to each judge record in our Wikipedia data set. In total, our scraped Wikipedia data comprises 3,140 former and current district judges, but after filtering the data for judges who were active from 2001 to present, 1,639 judges remain.

### Federal Judicial Center biographical directory of article III federal judges

An alternative source for information on federal district judges is a database maintained by FJC [30]. This database, called the Biographical Directory of Article III Federal Judges, provides a publicly-available.csv file where each row is the record for an individual judge. The record includes information about the judge’s demographics, federal judicial service, education, professional career, confirmation process, and more [30].

For consistency with the databases described above, we extract the CourtName field for each judge and convert it to the CIRCDIST numbering scheme for these judicial districts. Because a small number of judges serve for two districts in their state, we duplicate the rows for these judges and record the corresponding CIRCDIST for each entry. We also add variables for the overall starting and ending years of service of each judge, based on information recorded in the CommissionDate and TerminationDate fields in this data set. Finally, since the LastName, MiddleName, and FirstName are separate in this data set, we extract the initials from each name and concatenate them into an Initials field.

This data set of judges consists of 3,799 former and current district judges. After filtering the data for judges active after 2001, 1,634 judge entries remain. A difference between this FJC data set and the Wikipedia judges data set described above is that the FJC judge data set does not include judges from the three territorial courts since they are not governed by Article III of the Constitution. In addition, both the Wikipedia and the FJC judge data sets each contain entries of several judges (not included in the other data set) who have not yet been commissioned and will begin service in 2020. These judges will not appear in our final data set since our USSC data is restricted to fiscal years 2002-2018.

## Data consolidation

### Merging Federal Judicial Center integrated database

Each record in the USSC database has a unique identifier stored in the variable USSCIDN. However, this identifier is not used in the FJC Integrated Database. Therefore, to link information, we use an approach established in [6, 9, 10]. We perform a data merge on critical variables that are shared between these two data sets, namely federal judicial district, month of sentencing, year of sentencing, prison sentence given, fine given, and probation given. Within our preprocessed USSC data, this information is stored in the variables DISTRICT, SENTMON, SENTYR, SENTTOT, FINE, and PROBATN, respectively. Within our preprocessed FJC data, it is stored in SENTMON, SENTYR, DISTRICT, PRISTOT, FINETOT, and PROBTOT.

Records that have values of NA for any of these variables cannot be matched, and so we drop them from our data sets. These deletions reduce the USSC data from 1,265,688 records to 1,245,827 records, and they reduce the FJC data from 1,455,947 records to 1,452,288 records.

We then carry out a basic merge of the two data sets across the aforementioned six core variables, retaining only unique matches. That is to say, for each record in the USSC data, we check if there is exactly one record in the FJC data that has the same federal district, sentencing month and year, and prison, fine, and probation penalties. If a match is made, we merge the records. As an additional step to validate the matching, we require that for any merged record, at least one of the values of USSCcode1—USSCcode5 must be included in the values of FJDcode1—FJDcode10. That is to say, we check that the two data sets have at least some overlap in how they report the parts of the U.S. code relevant to the court case. After carrying out these steps, we successfully merge 658,891 records, leaving 586,936 USSC records unmatched.

Recall that in the USSC data, the prison sentence variable SENTTOT is real-valued while in the FJC data, the prison sentence variable is integer-valued. Therefore, for the remaining unmatched records in USSC, we repeat the merging steps described above, but using the variable roundSENTTOT rather than SENTTOT in the USSC data. This procedure produces an additional 145,237 matches for a total of 804,128 matches, leaving 441,699 USSC records unmatched.

We repeat the merging procedure yet again, but using the variable floorSENTTOT rather than SENTTOT. This procedure produces an additional 56,092 matches for a total of 860,220 matched USSC records. There remain 385,607 USSC records that we are unable to match.

### Merging PACER

Having merged USSC and FJC data, we now integrate judge’s initials from PACER to each case record. To understand how we perform this merge, it is important to remember two facts. First, the variable CASLGKY in our merged USSC/FJC carries, for our purposes, the same information as the pairing of district and docket number that we use to query PACER. In the discussion below, we explain our merging procedure using CASLGKY. Second, the basic units of data in USSC/FJC and in our PACER data are different. In USSC/FJC, each record describes the sentencing outcomes for an individual. However, criminal cases may contain more than one defendant, meaning that CASLGKY need not be unique within our USSC/FJC data. In fact, we find 86,251 values of CASLGKY that are not unique. The most frequently appearing one occurs 89 times, indicating a criminal case that had 89 different defendants (as verified through inspection of the defendant number variable, DEFNO). Equally, as mentioned before, searching for a particular case in PACER may return multiple records due to multiple defendants.

Thus, we merge the USSC/FJC data with PACER data in the following manner. First, we select a record from the USSC/FJC data. We search our PACER results for every record with the same value of CASLGKY. If we find that these PACER records all have the same judge initials, we assign these initials not just to the record we selected, but to any other record in our USSC/FJC data that shares the same value of CASLGKY. In other words, when there are multiple defendants, we are only able to assign judge initials to USSC/FJC records when all defendants are sentenced by the same judge. Otherwise, we are not able to assign judge initials, as there is no reliable to way to map a particular defendant in USSC/FJC to a particular defendant in PACER.

Our merging procedure produces the following results. We begin with the 860,220 records in our USSC/FJC data, which comprise 697,555 unique values of CASLGKY. In our 3,394,237 PACER records, there are 645,457 distinct values of CASLGKY. Of these, 524,393 are unique, and as a result, give us unique judge initials. An additional 106,436 values of CASLGKY appear more than one time in our PACER data but still have unique judge initials. Overall, when we merge our USSC/FJC data with our PACER data, we are able to assign initials to 809,405 of the 860,220 records in the former.

### Merging Wikipedia

Our next merging step involves matching the judges’ full names to their initials for each defendant case in the USSC/FJC/PACER data set. Recall that our scraped Wikipedia data includes each judge’s district and two versions of the judges’ inferred initials: one from the district table and another from their individual Wikipedia page, if available. Therefore, we merge the full names to initials by district. In particular, we use the variable CIRCDIST from USSC [17], which we have also added to the Wikipedia judges data set.

There are two primary cases to consider: first we match judges with initials that are unique within their district, and second, we match judges with initials that are not. Since our data set covers criminal cases after 2001, we identify only 15 duplicated judge initials out of 1,639 judge initials-district pairs. In addition, we also take into account the fact that Juriscraper queries return two sets of judge initials in the PACER full case title in 187,799 cases of our 809,405 records. For instance, the case title “1:00-cr-00425-RWR-AK USA V. Hill et all (closed 04/26/2002)” gives initials “RWR” and “AK” for federal judges (and potentially magistrates) assigned to this case. Before proceeding with the two merging stages accounting for each case, we separate the USSC/FJC/PACER data set into cases where judge initials correspond to unique judge initials in a district (comprising the vast majority of cases), and cases where they correspond to duplicated judge initials in a district.

In the first merging stage, we include only unique judge initials-district combinations from the Wikipedia data set. We first merge USSC/FJC/PACER data with the judges data based on the variables CIRCDIST and the first set of judge initials from PACER. This merge uses the variable judge_inits in the Wikipedia data set and yields 552,878 matches. We repeat the same merging process for the remaining unmatched cases but instead use the second form of the initials from the Wikipedia data set, judge_inits_full. This second merge yields an additional 47,629 matches. We then repeat the above steps for the remaining unmatched cases but instead use variables CIRCDIST and the second set of judge initials from PACER. As before, we first use the variable judge_inits in the Wikipedia data set, which yields 262 additional matches. We then use the variable judge_inits_full in the Wikipedia data set, which yields an additional 19 matches.

In the second merging stage, we separately match each duplicate judge initials-district combination in our Wikipedia data set with the USSC/FJC/PACER data set. Since there are two judges corresponding to one district-judge initials pair, we determine factors that allow us to distinguish between cases assigned to either of the judges. These factors include judge activity periods that do not intersect, availability of data only for one of the judges’ activity periods, and judges that have only served from 2019 on, a period which is not included in our data. In several duplicate cases, one of the judges has an additional middle name (provided in their individual Wikipedia page and stored in judge_inits_full), so we verify that they already were matched to USSC/FJC/PACER cases in the first merging stage. We then assign the remaining criminal cases associated with this judge initial-district combination to the second judge. There are several exceptions we encounter when dealing with these duplicate judge initials-district combinations. For instance, one of these combinations does not appear in our USSC/FJC/PACER data set. There is also one duplicate combination where we could find no distinguishing factor: there are two judges with initials “CAB” in the Southern District of California, Hons. Cathy Ann Bencivengo and Cynthia Ann Bashant, with largely overlapping activity periods. There is also a duplicate combination where we are only able to assign cases to each of the judges outside the intersection of their activity periods. Finally, we find an additional duplicate judge initials-district pair when considering the full judge initials from Wikipedia individual pages.

Overall, there are 600,788 matches of the USSC/FJC/PACER data set with judges from Wikipedia based on unique judge initials-district pairs and 6,985 additional matches from the duplicate judge initials-district combinations. By analyzing the cases that were not matched to judges in this merge, we identify eight districts for which none of the cases have matches to judges from our Wikipedia data set. In six of these districts (the Southern District of West Virginia, the Southern District of Texas, the District of the Northern Mariana Islands, the District of Guam, the Northern District of Illinois, and the Middle District of Tennessee), no judge initials are reported in PACER, making the connection to the judges’ identities impossible. In the Eastern District of North Carolina, almost 45% of the cases report only one letter for judge initials and therefore cannot be matched. The majority of the remaining cases have letter combinations that do not correspond to existing judge names. We thus conclude that the letters in this district’s docket numbers may correspond to other notes about the criminal case. Similarly, very few matches are possible for the Northern District of Texas, where almost 96% of the cases report only one letter for judge initials, and in the Western District of Oklahoma, where almost 83% of the cases report only one letter for judge initials. Finally, in the District of New Hampshire, we find that the judge initials reported in PACER do not include the judges’ middle initials. We address this issue by removing the middle initials from variable judge_inits_full and are able to match an additional 2,685 cases, corresponding to 99.2% of this district’s cases in our data set.

We complete an additional validation step by checking whether variable SENTYR in this final merged data set is within the assigned judges’ activity period. We find that 589,433 of the cases satisfy this requirement, and we accept these as the product of this stage of our work.

### Merging Federal Judicial Center biographical data

Rather than using Wikipedia to map judge initials to names, an alternative approach is to use information from the FJC biographical database. This approach has the advantage that the resulting data set contains additional information about federal judges. At the same time, a disadvantage is that judges from the three U.S. territories are excluded because they are not Article III judges. In addition, this data set only includes one set of judge initials. As in the case of merging with the Wikipedia data set, we also use the district variable CIRCDIST from USSC to merge the judge full names to their initials.

We restrict the data set to judges active after 2001 and identify 13 duplicates out of 1,634 judge initials-district pairs. These duplicate pairs are a subset of the 15 duplicate pairs we identified in the Wikipedia data set. As in the previous section, we consider separately judges whose initials are unique within their district and those whose initials are not.

In the first merging stage we include only unique judge initials-district combinations from the FJC judge data set. We merge USSC/FJC/PACER data with the judges data based on the variables CIRCDIST and the first set of judge initials from PACER, which yields 584,000 matches. We then repeat this merge for the remaining unmatched cases but instead use variables CIRCDIST and the second set of judge initials from PACER. This process yields 241 additional matches.

In the second merging stage, we separately match each duplicate judge initials-district combination in our FJC judge data set with the USSC/FJC/PACER data set, restricted to cases where judge initials are duplicated in a district. Since the duplicate judge initials-district combinations in this data set are a subset of the ones identified in the Wikipedia data set, we use the same factors described in the previous section to distinguish between cases assigned to either judge.

Overall, we find 584,241 matches of the USSC/FJC/PACER data set with judges from the FJC biographical data based on unique judge initials-district pairs, and 6,196 additional matches from the duplicate judge initials-district combinations. As in the previous section, we treat matches for the District of New Hampshire separately, since judge initials reported in PACER do not include middle name initials. By removing the middle initials from variable Initials in the FJC judge data set, we are again able to match an additional 2,685 cases to judges in this district.

To validate the combined matched data set, we check whether variable SENTYR is within the corresponding judges’ activity period. We find that 573,171 of the cases satisfy this requirement, and therefore these cases are the final product of this merging step.

### Finalizing JUSTFAIR

We combine the results of the previous two merging steps to provide a final data set that links cases and defendant information to judge information from both the Wikipedia district court pages and the FJC biographical data. In comparing the matching of each USSC/FJC/PACER defendant case to judges’ full names and characteristics using Wikipedia versus FJC judge data, we find only 12 discrepancies. These discrepancies all come from cases where PACER records contain two sets of initials; see PACER and Juriscraper. For example, a case in the Eastern District of Michigan is assigned initials “AC-LJM” in PACER. The first set of judge initials, “AC,” corresponds to Hon. Avern Cohn (as recorded in Wikipedia). In the FJC biographical data, this judge is recorded as Avern Levin Cohn, and therefore the merge cannot be done using the “AC” initials stored in pacer_first_inits. Instead, this case gets merged to Laurie Jill Michelson. Since pacer_first_inits is more likely to correspond to the federal judge assigned to the case, we removed 8 cases from the data set merged with Wikipedia and 4 cases from the data set merged with FJC, which correspond to merges using the second set of judge initials, pacer_second_inits.

Of the 589,433 cases matched with judges from Wikipedia, 22,683 cases are not matched to any judge in FJC. These cases correspond to 45 judges, 3 of whom serve on the District Court of the Virgin Islands. These three judges cannot be found in the FJC data, which only lists Article III federal judges. For the remaining 42 judges, the initials extracted from Wikipedia are consistent with how these judge initials are recorded in PACER; however the initials extracted from FJC for those judges are not. This inconsistency can occur because of a missing middle name, additional middle name, different writing of the last name, or similar issues. For example, a federal judge in the Eastern District of Pennsylvania is recorded in Wikipedia as Hon. Franklin Van Antwerpen, whereas in the FJC biographical data their name is recorded as Franklin Stuart Van Antwerpen. Similarly, of the 573,171 cases matched with judges from FJC, 6,425 cases are not matched to judges in Wikipedia. All these judges have slight differences in initials as compared to the same judges in the Wikipedia data set.

We assemble the final data set so that each row provides the case, sentencing, and defendant information, as well as judge information extracted from both Wikipedia and from the FJC biographical data (when available). This combined data set contains 595,850 cases and 557 variables. We add a final variable (column) called DataSource to this data set, which takes the value 1 if the case is matched with both judge data sources, value 2 if the case is matched with the Wikipedia court page judge source only, and value 3 if the case is matched with the FJC biographical data judge source only.

## Data quality and validation

As described in previous sections, in the creation of our JUSTFAIR data set, we take several measures to ensure data quality. When merging the USSC and FJD databases, we validate merged records by checking for common offense codes. When assigning names of judges to sentencing records based on initials, we use two independent sources. And finally, we require that the date of sentencing for a case fell within the time period during which the inferred judge was active.

We now perform a validation procedure on our data in order to estimate accuracy. We measure the accuracy of judge inferences using bulk data available from the FreeLaw CourtListener project [28]. CourtListener provides free, archived access to the text of 3.6 million judicial opinions collected from PACER. To create our validation set, we randomly sample CourtListener records for which the docket number includes the string “CR,” suggesting a criminal case, and for which there is an identified judge in the record. After using a small number of records to iteratively fine-tune our validation process and data pipeline, we randomly select 400 records to use as a validation set. In the discussion below, it is important to note that judicial opinions are typically not directly associated with criminal sentencing proceedings. Instead, judicial opinions are associated with procedural issues that occur during the case, such as whether certain evidence is admissible. Thus, an underlying assumption of our judge identification procedure (which we will see is occasionally violated) is that the judge involved in a proceeding is the same as the sentencing judge.

To validate based on a CourtListener record, we first search our scraped PACER results for a record with a matching district and approximately matching case title. If we obtain such a match, we can infer a CASLGKY identifier which then allows us to locate the case in JUSTFAIR. However, there are many reasons that a sampled CourtListener record might not be found in JUSTFAIR. It is possible that the information about the case could not be unambiguously matched during one of our merging steps, and hence is not included in our final data. Alternatively, the record sampled from CourtListener might in fact not be a criminal case despite the appearance of the string “CR” in its docket number. Or, despite the proceeding being a criminal matter, we may not be able to locate a sentencing associated with it. Finally, the CourtListener record might be related to a sentencing that is outside the time period of our study.

For the CourtListener records for which we can locate a related record in JUSTFAIR, we check whether the CourtListener judge for the procedural opinion matches the JUSTFAIR judge for the criminal case associated with the procedural opinion. If there is a match, we accept our JUSTFAIR record as correct. If there is not a match, we attempt to use alternative methods including PACER searches and news searches to see if the JUSTFAIR judge might nonetheless be correct. For instance, a minor procedural matter in a criminal case might be handled by a magistrate judge who would be listed in the CourtListener record, and yet the sentencing decision would be made by a full-fledged district judge.

We sample 400 records from CourtListener. Of these, 243 do not appear in JUSTFAIR for the reasons mentioned above. For the 157 CourtListener validation cases that do appear in JUSTFAIR, JUSTFAIR has the correct judge for 154 of them. Our three incorrect cases merit some discussion.

One of our incorrect inferences pertains to *United States v. Christy* from the District of New Mexico. The sampled record is an opinion for a procedural matter related to suppression of evidence in a child pornography and sexual exploitation trial. The opinion was authored by Hon. James O. Browning, and a search of the news indicates that he was, in fact, the sentencing judge. In JUSTFAIR, we have listed the judge as Hon. James Aubrey Parker. Parker was indeed involved with this criminal case, but only in post-conviction proceedings that occurred several years after sentencing. Thus, our inference is incorrect.

A second incorrect inference is for *United States v. Rajaratnam*, a high-profile securities fraud case from the Southern District of New York. The sampled record is an opinion from a post-conviction proceeding in which the defendant moved for acquittal on procedural grounds. The opinion was authored by Hon. Richard J. Holwell, and a news search confirms that Holwell was the sentencing judge. However, in JUSTFAIR, we have listed the judge as Hon. Loretta A. Preska, who was involved with the case only in post-conviction proceedings some years later. This is an error similar to the one discussed above for the *Christy* case.

Finally, our third incorrect inference is for *United States v. Council*, a firearm possession case from the Eastern District of Virginia. The judge listed in CourtListener is Hon. James R. Spencer, who, indeed, was the sentencing judge. JUSTFAIR, however, infers the judge to be Hon. Henry E. Hudson. As with the previous two incorrect cases, Hudson became involved with the case several years after sentencing.

In summary, we sample 400 cases from CourtListener and find 157 of them in JUSTFAIR. Of these 157, we correctly infer the sentencing judge for 154 of them. The three incorrect cases (which we have manually corrected in our data) all correspond to situations where a judge other than the sentencing judge became involved in proceedings post-sentencing. Overall, our measured case-to-judge accuracy rate is 98.1%. A standard statistical calculation yields that a 95% confidence interval for the accuracy rate within the entire JUSTFAIR data set is 94.5% to 99.6%.

## Conclusion

With nearly 600,000 records, JUSTFAIR is the first large scale, free, public database that links information about defendants and their demographic characteristics with information about their federal crimes, their sentences, and crucially, the identity of the sentencing judge. We have inferred the sentencing records in JUSTFAIR by using data from the United States Sentencing Commission, the Federal Judicial Center, the Public Access to Court Electronic Records system, and Wikipedia. We have performed manual data checks to validate our inferences of sentencing judge, and we estimate 98% accuracy within our data set.

We remind the reader of some limitations of our data. First, merging information from the United States Sentencing Commission and the Federal Judicial Center requires matching the federal judicial district and sentencing date of a case and the prison time, probation, and monetary fine imposed on the defendant. This matching is possible, then, only when the aforementioned combination of variables is unique. Second, our judge identification procedure depends on strings of letters obtained from querying PACER. If those letters are not available, or if they do not represent judge initials, we cannot infer a judge. As discussed previously, the result is that JUSTFAIR does not contain cases from the Southern District of West Virginia, the Southern District of Texas, the Northern District of Illinois, the Middle District of Tennessee, the Eastern District of North Carolina, the District of the Northern Mariana Islands, and the District of Guam, and contains limited results for the Northern District of Texas and the Western District of Oklahoma.

The United States Constitution guarantees the public’s right to access criminal court proceedings. At present, the judicial branch releases criminal case data in a manner that obfuscates the identity of sentencing judges. Perhaps ironically, our two primary data sources, the United States Sentencing Commission and the Federal Judicial Center, are physically co-located in the Thurgood Marshall Federal Judiciary Building in Washington, D.C. Before JUSTFAIR, the information necessary to identify sentencing judges lived behind a paywall and was therefore inaccessible to many people. We have made the JUSTFAIR data set public [31] and we have created an interactive online visualizer for exploration of the data [32]. By doing so, we hope to correct the inequity of access to information about sentencing judges.

While the government has chosen to release case data piecemeal, the JUSTFAIR project demonstrates that it is possible to put the pieces back together. Given this capability, we encourage the government simply to release full data with identified judges. Doing so would provide the public with a more complete and accurate data set than JUSTFAIR, resulting in increased transparency.

Beyond providing insight into our federal criminal justice system, JUSTFAIR enables a more granular examination of federal sentencing disparities than has previously been possible. Moving forward, we anticipate analyzing district level and individual judge level data to assess the presence or absence of sentencing disparities, especially along racial lines.
